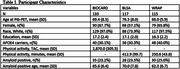# Physical activity and amyloid onset age: Findings from three studies of community‐dwelling adults

**DOI:** 10.1002/alz.091417

**Published:** 2025-01-09

**Authors:** Ryan J. Dougherty, Jiangxia Wang, Murat Bilgel, Tobey J. Betthauser, Susan M. Resnick, Ozioma C Okonkwo, Sterling C. Johnson, Jennifer A Schrack, Marilyn S. Albert

**Affiliations:** ^1^ Johns Hopkins University, Baltimore, MD USA; ^2^ National Institute on Aging, National Institutes of Health, Baltimore, MD USA; ^3^ University of Wisconsin‐Madison School of Medicine and Public Health, Madison, WI USA; ^4^ Johns Hopkins Bloomberg School of Public Health, Baltimore, MD USA

## Abstract

**Background:**

Higher levels of physical activity, measured through accelerometry, have shown associations with several indices of brain and cognitive health in middle‐to‐late aged individuals. The association of physical activity and AD‐specific biomarkers measured through positron emission tomography (PET), however, remains unclear. Previous studies show no direct association of physical activity with amyloid‐PET levels, but associations with the onset of amyloid positivity remain unexplored. This study used estimated amyloid onset age (EAOA), defined as the age at which a participant is expected to attain the amyloid positivity threshold based on longitudinal PET, to investigate if higher levels of physical activity are associated with later EAOA across three cohort studies including: the Biomarkers of Cognitive Decline among Normal Individuals (BIOCARD) study, the Baltimore Longitudinal Study of Aging (BLSA), and the Wisconsin Registry for Alzheimer’s Prevention (WRAP) study.

**Method:**

Cognitively unimpaired participants from BIOCARD (N = 133), BLSA (N = 117), and WRAP (N = 115) who completed accelerometer‐measured physical activity monitoring over 7 days and underwent ^11^C‐Pittsburgh compound B PET imaging were included. Total daily physical activity was measured through wrist‐worn (BIOCARD, BLSA) or waist‐worn (WRAP) accelerometers and EAOA was estimated using sampled Iterative Local Approximation. Proportional Hazard Cox regression models assessed whether physical activity is associated with EAOA, using right‐censoring for participants who did not develop amyloid positivity, adjusting for sex and *APOE* status. Potential interactions between physical activity and *APOE* status were also assessed.

**Result:**

Participant demographics are displayed in Table 1. Across all three cohorts, *APOE* status was associated with EAOA (all p<.05), such that *APOE*‐e4 carriers had an earlier amyloid onset. We did not observe associations between physical activity and EAOA among BIOCARD (z‐score HR = 1.27, p = 0.27), BLSA (z‐score HR = 1.01, p = 0.97), or WRAP participants (z‐score HR = 0.96, p = 0.85). Furthermore, no significant interactions between physical activity and *APOE* status were observed (all p>.05).

**Conclusion:**

Across three separate studies of community‐dwelling middle‐ age and older adults, we did not observe an association between accelerometer‐measured physical activity and EAOA. Further research is needed to i) determine if alternate methods for assessing physical activity are associated with AD biomarkers and ii) investigate non‐AD pathways through which physical activity may impact cognitive function and AD risk.